# Rearing European Eel (*Anguilla anguilla*) Elvers in a Biofloc System

**DOI:** 10.3390/ani13203234

**Published:** 2023-10-17

**Authors:** Luis Vinatea, Ricard Carbó, Karl B. Andree, Enric Gisbert, Alicia Estévez

**Affiliations:** 1 Departamento de Acuicultura, Universidade Federal de Santa Catarina (UFSC), Florianópolis 88061-600, SC, Brazil; 2 Aquaculture Program, Centre de la Ràpita, Institut de Recerca i Tecnología Agroalimentàries (IRTA), Ctra. Poble Nou, km 5.5, 43540 Sant Carles de la Rapita, Spain; ricard.carbo@irta.cat (R.C.); karl.andree@irta.cat (K.B.A.); alicia.estevez@irta.cat (A.E.)

**Keywords:** European eel, biofloc, sustainable aquaculture, biofloc microbiota

## Abstract

**Simple Summary:**

Sustainable strategies for improving blue food economies are essential in order to design a new approach to transitioning towards more responsible, comprehensive, exploitable production and consumption models that have a positive impact on society and the environment. Among different fish farming systems, biofloc technology (BFT) is considered one of the most cost-effective, sustainable, and environmentally friendly farming systems due to its zero-water exchange and improvement of feed conversion ratio to the dietary contribution of bioflocs growing in the system, which contribute to the nutrition of the farmed aquatic animal. Thus, a two-month trial was conducted to evaluate the feasibility of rearing European eel (*Anguilla anguilla*) elvers, a species that is generally farmed in recirculation aquaculture systems, using BFT.

**Abstract:**

European eel (*Anguilla anguilla*) elvers (initial body weight (BW) = 3 g) were raised in triplicate for 60 days in a biofloc system (BFT) at 21 °C. Data from the current first study evaluating this farming technology indicated that European eel elvers adapted well to BFT systems as data on growth performance (specific growth rate = 1.48% ± 0.13 BW/day and FCR = 1.05 ± 0.09) indicated, with production costs using BFT being lower than conventional RAS units. The most critical issues associated with this aquaculture system were the maintenance of the biofloc in tanks by the regular addition of refined sugar (46% C) to keep a relationship for C:N of 20:1, and the prevention of emergence of opportunistic pathogens like the monogenean *Pseudodactylogyrus* sp. The overall results of this study in terms of elvers’ performance and quality and the composition of the biofloc material and its microbial composition indicated that BFT, which is considered to be one of the most cost-effective, sustainable, and environmentally friendly farming systems due to its zero water exchange and improvement of feed conversion ratio by the dietary contribution of bioflocs, may be satisfactorily used for farming European eels elvers at a density of 2 kg/m^3^. However, further studies are needed to test this technology with older eel stages.

## 1. Introduction

The European eel *Anguilla anguilla* (Linnaeus, 1758) is a fishing resource of great economic and cultural importance in most parts of Europe and North Africa [1,2]. Its exploitation depends entirely on the capture of wild glass eels during the migratory process from marine to continental waters [3,4]. Its culture, both in ponds (extensive) and in recirculation (intensive) systems, started in 1985 in Holland, Italy, Denmark, Spain, and Greece, mostly in recirculating aquaculture systems (RAS). At present, the production of eel meets the demand of the European and Asian markets. However, a significant expansion of the industry is not expected due to the species being threatened with extinction [5,6].

Important research efforts have been made in recent years to control the reproduction [7,8,9] and larval rearing [10,11] of the genus Anguilla. In the case of on-growing, RAS technology seems to be the main technology used worldwide [6,12,13]. The production of eels in RAS was developed to save energy and reduce water treatment costs [14]. Animals are kept in densities higher than 120 kg/m^3^ in indoor tanks with strong water flow to provide enough oxygen and to remove metabolites such as ammonium and carbon dioxide as well as faeces and feed residues. While the technical sophistication of such systems can save water and energy, the system demands highly specialized workers and a team of experts capable of operating complex facilities. Additionally, it requires a relatively high investment due to the automation, equipment, and systems for the minimization of residues [15,16]. For these reasons, the search for cheaper, less complex, and more independent culture techniques, in terms of water and energy consumption, seems justified.

Among current aquaculture technologies, those that contribute the most to the preservation of natural resources in terms of water use and minimal renewal are (1) recirculating systems (RAS) [15,16], and (2) microbial biofloc systems (BFT) [17,18]. As described by Crab et al. [19], in BFT systems, the water exchange does not occur as in the RAS, although a small part circulates through decanters of solids, and some water is added to compensate for losses due to evaporation. The transformation of ammonium is achieved through the addition of organic carbon to stimulate the growth of heterotrophic bacteria. Adequate oxygen concentrations are maintained by both mechanical aeration and pure oxygen injection. An advantage that BFT cultivation has is the possibility of microbial biomass being used as a nutritional supplement, resulting in the use of feed with lower protein concentration [20]. This is of special relevance since feeding costs in fish farms may represent up to 50–70 percent of production costs [21]. Different studies postulated that BFT can become a sustainable alternative to conventional recirculation systems, which require high protein feed and sophisticated filtration equipment, as well as complex automation systems [14,22] and elevated energy consumption [23,24]. In this context, sustainable strategies for improving blue food economies are essential to designing a new approach to transitioning towards more responsible, comprehensive, exploitable production and consumption models with a positive impact for society and the environment [25].

Among different rearing systems for on-growing eels, BFT may be a sound and sustainable alternative since biofloc systems are able to maintain water quality and substantially reduce its consumption, improve feed conversion ratios, and reduce production costs, and they may replace conventional high-cost feeds with alternative protein sources, among other technical benefits [19]. Thus, the objective of the present study was to verify for the first time the feasibility of growing European eel (*A. anguilla*) elvers in a microbial biofloc system with minimal water renewal and evaluate their performance in comparison to the conventional RAS where this species is generally reared. Such evaluation was conducted in terms of different biological parameters such as elvers’ condition and performance, the nutritional quality of the biofloc particles, and the economic impact of this technology in terms of labour, power, and feeding costs.

## 2. Materials and Methods

### 2.1. Animals and Experimental Design

Wild glass eels of *A. anguilla* were purchased from the private company “Angulas y Mariscos Roset S.L.” (Deltebre, Tarragona, Spain). These animals were captured during their onshore migration to estuarine and freshwater environments. Glass eels were transported by road (1 h trip) in a 500 L tank and acclimatized and kept at IRTA (Institut de Recerca i Tecnología Agroalimentàries) in la Ràpita (Tarragona, Spain) where the current trial was conducted. During this acclimation period, fish were kept as described in Gisbert et al. [26] and progressively weaned onto an inert compound feed (“Perle Eel proactive”, Skretting; 54% crude protein and a pellet size of 0.7 mm diameter); thus, elvers were able to accept the former aquafeed at the beginning of the trial when elvers were 2.99 ± 0.97 g (mean ± standard deviation) in initial weight (BW). Elver rearing in BFT was evaluated in triplicate (n = 3 BFT tanks). A total of 1350 European eels at elver stage were equally distributed among experimental 1000 L conical fiberglass tanks (450 specimens per tank) at an initial density of 1.3 kg/m^3^. For this purpose, all animals were anesthetized with tricaine methanesulfonate (100 mg/L; MS222, Sigma-Aldrich, Alcobendas, Spain) and individually weighed and measured in total length to the nearest 0.1 g and 1 mm, respectively, in order to guarantee similar stocking biomass among experimental tanks and avoid fish size hierarchy and potential cannibalism. Elvers with external lesions and redness of paired and unpaired fins were excluded from the trial. Elvers were fed at 4% of stocked biomass five times per day from 08 h to 18 h by means of automatic feeders (EHEIM Autofeeder, EHEIM GmbH & Co. KG, Deizisau, Germany). The photoperiod was 12 h light and 12 h darkness. The trial lasted 60 days.

### 2.2. The BFT System and Water Quality Parameters

The biofloc inoculum used in the current trial was obtained from a parallel BFT trial run on tench (*Tinca tinca*) [27]. Each BFT tank was inoculated with 100 L of the above-mentioned inoculum and 900 L of fresh water, previously treated and filtered (water obtained from an artesian well 40 m deep with an electrical conductivity of 2500 μS/cm). To promote biofloc growth, the activity of heterotrophic bacteria was stimulated with the daily addition of common refined sugar (46% C). The criterion recommended by Avnimelech [28] was followed to calculate the amount of carbohydrates to be added into each BFT tank, in which a relationship C:N of 20:1 is recommended. Decanters of solids with a usable volume of 100 L were coupled to the cultivation tanks and operated through an airlift when the total solid concentration exceeded 500 mg/L.

**Figure 1:**
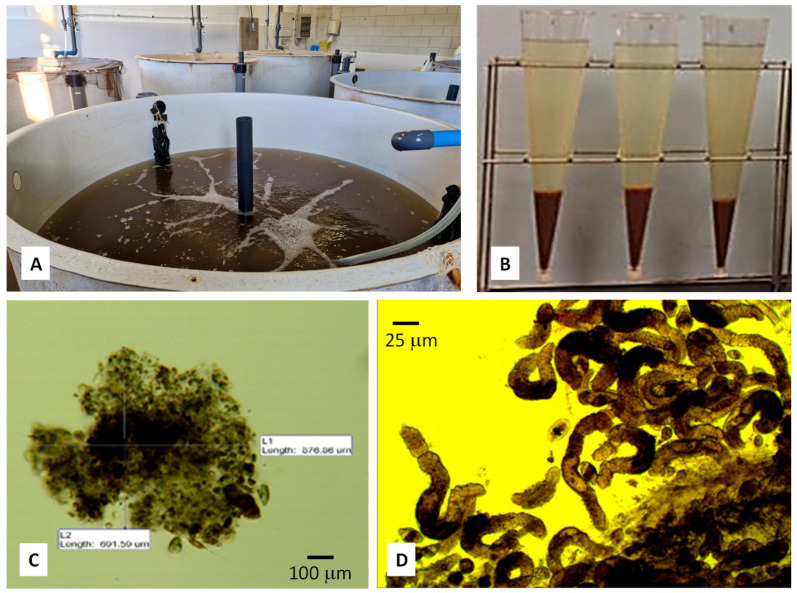
General view of the experimental BFT tank showing the muddy colour of water due to the growth of biofloc particles and the strong aeration provided to keep biofloc particles in suspension in the water column (**A**). View of the Inhoff cones used for regular evaluation of suspended solids in the system (**B**). Detail of a biofloc particle under the binocular microscope for its measurement (**C**). Detail of a biofloc particle in which nematodes attached to the organic matter of the biofloc may be seen, and some isolated protozoans may be identified (**D**).

Water quality parameters such as temperature, dissolved oxygen, pH, total ammonia, and nitrite were daily monitored, while total suspended solids (SST) were determined every two days. Alkalinity and nitrate were controlled once per week (Table 1). Measurements of suspended solids were made using the gravimetric method, which measures total suspended solids and settleable solids within an Imhoff cone [27]; for this purpose, samples of water (2 L) were poured into the Imhoff cone, and suspended solids were allowed to sediment for 25 min (Figure 1). Samples of biofloc particles were observed under a binocular microscope (Nikon SMZ 800, Nikon Instruments Inc., Melville, NY, USA) under different levels of magnification (40, 100, and 200×) and measured using an image analysis software package (ANALYSIS; Soft Imaging Systems GmbH, Münster, Germany). Biological groups in bioflocs were identified at the highest taxonomical level. As ammonia is a critical parameter in BFT systems, total ammonia was determined by two different methodologies: once a day with Merck Colorimetry Kits (MColortest^TM^; Merck KGaA, Darmstadt, Germany) and once per week through the analytical method of indophenol [29] using microplates (Infinite M200 spectrophotometer; Tekan trading AG, Männedorf, Switzerland). Colorimetric values were correlated with those of the indophenol method in order to generate an equation capable of correcting the subjectivity of the colorimetric method that it is generally used in aquariology.

### 2.3. Zootechnical Parameters

Every 15 days, 50 juveniles were randomly sampled, gently anesthetized with MS222 as previously described, and weighed to the nearest 0.1 g in order to check their growth rate and to readjust the amount of food to be offered based on the estimated stocked elvers’ biomass. At the end of the experiment, 100 individuals from each experimental BFT unit were netted and anesthetized as described, and their body weight and total length (TL) were measured to the nearest 0.1 g and 0.1 cm, respectively. Ingesta of pellets of the compound feed could not be directly measured in BFT tanks due to water turbidity (high levels of TSS) and the strong water aeration; thus, for determination of the feed conversion ratio, the amount of distributed feed was only used for calculation, and consequently, we determined the apparent feed conversion rate (FCRa). Similarly, it was not possible to assess the level of ingesta of biofloc particles by European eel elvers to determine their potential contribution to elvers’ growth performance.

The following standard formulae were used to evaluate European eel elver’s key performance indicators when reared under BFT:
Specific growth rate (SGR, % BW/day) = [(ln BW_final_ − ln BW_initial_) × 100]/time] × 100,Fulton’s condition factor (K) = (BW × 100)/TL^3^,Apparent feed conversion rate (FCRa) = amount of compound feed administered/fish biomass gain.Survival (S, %) = (N_final_/N_initial_) × 100,
where BW_final_ and BW_initial_ are elvers’ body weight at the end and beginning of the trial, and N_final_ and N_initial_ are the number of elvers at the end and at the beginning of the trial, respectively.

The monthly cost assessment of BFT units was computed using daily data of electricity costs (air blowers for BFT), elvers’ feed distribution, sugar administered into the tanks to promote biofloc growth, and personnel labour. The costs for rearing elvers in RAS units were estimated from a similar previous trial run in our facilities [30]. Other potential factors like the technological readiness level of BFT or needs for the capacitation of operators, among others, were excluded since they may largely change depending on the geographical area where this farming technology might be implemented.

### 2.4. Microbiota Diversity Analyses

To assess the effect of BFT on the microbiome in the water and the elver intestinal samples, the restriction fragment length polymorphism (RFLP) technique was used as a proxy. The microbiome in the elver gut and water samples from a RAS unit were sampled from a similar number of elvers as those sampled from the BFT unit, since we kept a group of elvers in a 2000 L tank connected to a RAS unit (IRTAmar^TM^) as a back-up. For this purpose, *A. anguilla* elvers (n = 5) were collected from each tank (BFT and RAS units) and euthanised with an overdose of anaesthesia (400 ppm MS222), and their entire intestine was aseptically dissected and immediately fixed in 70% ethanol and stored at 4 °C. Prior to DNA extraction, tissue samples were washed with buffered peptone water to remove traces of ethanol. Then, the tissue was minced into small pieces using sterile scissors and then placed into a 15 mL tube with a small aliquot of zirconium glass beads (1.0 mm diameter, BioSpec Products, Bartlesville, OK, USA). The 15 mL tube was shaken by hand vigorously for 3–5 min until a uniform homogenate was obtained. A volume of 400 µL of this homogenate was used for DNA extraction.

Furthermore, at the end of the experiment, water samples were also collected from the tanks, as was a sample of the biofilm/sludge material from the BFT tanks. Bacterial sludge from the biofilm was collected into a pellet by centrifugation, and the supernatant was removed prior to DNA extraction. The water samples were filtered using 0.2 μm membrane filters, and the filters were cut into small strips to fit into a microcentrifuge tube for DNA extraction. A DNA Stool Mini Kit (Qiagen, Hilden, Germany) was used for DNA extraction from all samples according to the manufacturer’s instructions. The purity of extracted DNA was evaluated by means of spectrophotometry (GeneQuant, Amersham Biosciences, Piscataway, NJ, USA) utilizing the ratio of absorbance at λ = 260/280 nm to confirm the absence of residual protein content. A fragment of approximately 600 bp (size varied with taxa) of the 16S rDNA from total bacteria was amplified in a volume of 50 µL using primers previously described [31,32]: 5′-CTACGGGAGGCAGCAGT-3′ and 5′-CCGTCWATTCMTTTGAGTTT-3′. Each reaction included 100 ng of DNA and had a final concentration of 2 mM MgCl_2_, 1 mM dNTP’s (0.25 mM each), and 0.2 mM of each primer. Amplification conditions were 94 °C for 4 min followed by 35 cycles of 94 °C for 1 min, 45 °C for 1 min (with an increase of 0.1 °C each cycle), followed by 72 °C for 1 min 15 s. The program ended with a final extension step (5 min at 72 °C). After amplification, from a total of 50 µL of PCR solution, there were 5 different restriction enzyme digestions performed using Alu I, Hha I, Hpa II, Rsa I, and Sau 3AI (New England Biolabs, Ipswich, MA, USA). Each restriction enzyme digestion contained 6 µL of amplified 16S rDNA and an equal volume of digestion premix containing 5 units of enzyme and 2× reaction buffer. Each was mixed and incubated for 3 h at 37 °C. Reactions were stopped by incubation at 80 °C for 20 min. The restriction digests (12 µL) were run on a 2% agarose gel at 65 V/cm for 1 h. The final gel image was analysed using GeneTools (SynGene, Syngene International Ltd., Bengaluru, Karnataka, India).

### 2.5. Proximate Composition and Lipid Class of Bioflocs

The proximate composition and lipid classes of bioflocs growing into BFT tanks was assessed to evaluate their potential contribution to elvers’ nutrition as described in Vinatea et al. [27]. In brief, bioflocs were freeze-dried for 24 h and kept at −20 °C. Then, bioflocs were diluted in distilled water (10 mL), homogenized for 5 min with an Ultraturrax T-25 (IKA^®^ WERKE, Germany), and sonicated for 1 min (Vibra-cell, Sonics & Materials Inc., Newton, CT, USA). Crude protein and carbohydrate levels in bioflocs were determined according to the methods of Lowry et al. [33] and Dubois et al. [34], respectively. Samples for the protein analysis were previously digested with NaOH (40 mg/m/L at 60 °C for 30 min). Total lipids were extracted in chloroform: methanol (2:1, *v*:*v*) according to the Folch et al. [35] method. Furthermore, lipid class analysis was performed by high-performance thin-layer chromatography as described in Olsen and Henderson [36]. Bands were identified by charring the plates at 100 °C for 30 min after spraying with 3% (*w*/*v*) aqueous cupric acetate containing 8% (*v*/*v*) phosphoric acid and quantified by scanning densitometry using a GS 800 Calibrated Densitometer (Bio-Rad, Bio-Rad Laboratories, Inc., Hercules, CA, USA). All biochemical determinations for each sample were conducted in triplicate (methodological replicate).

### 2.6. Statistical Analyses

Data are presented as mean ± standard deviation. Changes in the proximate biochemical and lipid class composition of biofloc particles along different sampling points (days 4, 32, and 60) were analysed by means of a one-way ANOVA, with prior confirmation of normality and homogeneity of variance for selected values. Data expressed as percentages were arcsine-transformed prior to the ANOVA analysis. The potential stress derived from the position of the replicate BFT tanks in the experimental set-up on elvers’ growth performance, condition, and survival was explored by means of linear regression (*y* = a*x* + b), with *y* being a dependent variable (body weight, total length, SGR, K, and survival) and *x* the stress coefficient (independent variable) derived from the situation of the tank in the experimental design.

## 3. Results and Discussion

All recorded water quality parameters (Table 2) had stable values and remained within the range considered appropriate for most freshwater species [37,38], including European eel elvers [26,39].

Regarding total ammonia, two analytical methods were used in the current study (the colorimetric kit vs. the indophenol analytical method). In particular, the correlation made between concentrations of total ammonia recorded with the colorimetric kit and the analytical method showed that the kit overestimated 0.35 ± 0.26 mg NH₄⁺/L (from 0.05 to 1.14 mg/L) and underestimated 0.63 ± 0.31 mg NH₄⁺/L (from 0.04 to 1.15 mg/L). Despite the discrepancy, the use of the kits was useful due to the large amount of analysis that needed to be performed daily. The ammonia data in Table 2 corresponded to the values adjusted by the equation *y* = 0.8295*x* + 0131 (*R^2^* = 0.86; *p* < 0.05), where “*y*” is equivalent to the total ammonia corrected and “*x*” to the total ammonium recorded by the kit. In BFT farming systems, ammonia is generally controlled by the application of several sources of organic carbon [40]. BFT systems are reputed for their capacity to remove the nitrogenous waste through nitrification and denitrification processes; thus, the microbial communities of the bioflocs prevent the accumulation of toxic nitrogenous compounds for organisms [41]. Under current experimental conditions, the daily application of sugar in tanks maintained the concentrations of total ammonia (1.31 ± 0.98 mg NH_4_^+^/L) and of nonionized ammonia (0.039 ± 0.029 mg NH_3_/L) below the values considered dangerous for this euryhaline species [42]. In fact, Abbink et al. [43] reported a slight decrease in the growth performance of *A. anguilla* elvers (5 g) when exposed to 0.168 mg of NH_3_/L, even though such a decrease in somatic growth was not accompanied by an increase in fish mortality. In this sense, the low ammonia levels found in each of the three BFT tanks coupled to minimal changes in water pH and alkalinity values, along with the lack of accumulation of nitrates (Table 2), the final product of the autotrophic nitrification process, indicated that under current experimental conditions, ammonia control was primarily achieved through the heterotrophic pathway, guaranteeing proper water quality in terms of the level of this nitrogenous compound [44,45]. In this sense, all the solids remain in the BFT tanks as well as the organic carbon and nitrogen from the feed and faeces that are available for heterotrophic bacterial production. As Eveling et al. [44] described, since the energetics of heterotrophic bacteria are more favourable than those for autotrophic bacteria, it is assumed that the heterotrophic bacteria will first consume the available nitrogen using the readily available, labile carbon from the feed and faeces. The control of ammonia by the heterotrophic pathway in BFT systems considerably increases the production of suspended solids, mainly due to the increase in heterotrophic bacterial biomass since heterotrophic bacteria produce up to 40 times more biomass than nitrifying bacteria [44]. The low levels of nitrites (0.046 ± 0.06 mg/L) in BFT tanks, which never reached values close to toxic levels for European eel [46], indicated that autotrophic nitrification also took place but at a much lower magnitude than in the heterotrophic pathway [44,45]. However, its proportion in relation to aerobic heterotrophic bacteria was not determined in the current study.

Bioflocs underwent a significant morphological transformation throughout the experiment. At the beginning, the particles were small and consisted mainly of organic matter (faeces and feed residues), bacteria, and protozoa. After 15 days of trial, bioflocs were colonized by filamentous bacteria, whose effect resulted in an increase in the volume of the particles, change in colour (from brown to black), and rapid sedimentation in the Inhoff cones. In the middle of the rearing period, in addition to the microorganisms mentioned, bioflocs were colonized by rotifers *Brachionus* spp., whereas nematodes and many annelids *Aeolosoma* spp. (Polychaeta, Aeolosomatidae) were also found in biofloc particles (Figure 1).

**Figure 2:**
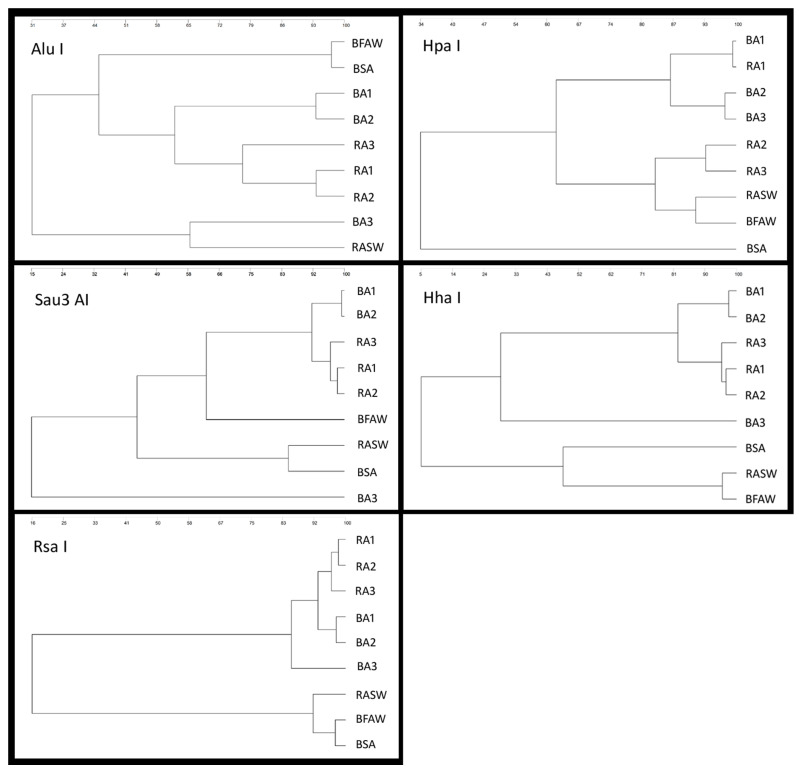
Dendograms (UPGMA method, GeneTools) from RFLP analysis of microbial samples collected from fish intestine, water, and biofilm samples in BFT and RAS systems. Each restriction enzyme used is shown in the upper left corner of the corresponding dendogram. BA = *A. anguilla* from biofloc tanks; RA = *A. anguilla* from RAS; BSA = biofloc sludge from tanks containing *A. anguilla*; RASW = water sample from RAS tank; BFAW = water sample from biofloc tank containing *A. anguilla*.

The RFLP analysis used as a proxy for the bacterial communities resident in the sludge and water from the tanks indicated that they resembled each other, therefore forming clades (Figure 2), whereas they differed from those in the intestine of elvers in either culture system. These results were expected, since the environment in the water (or sludge that collects in the water) is not as enriched as the gut of a fish, where nutrients accumulate and are degraded by enzymes for easier bioassimilation. The gut also produces substances that directly benefit microbial growth of some species (e.g., mucin proteins), while also producing substances that impair the growth of other species (e.g., antimicrobial peptides) [47]. The biofilm sludge material was the primary feed item and therefore a primary source of microbiota for colonizing the fish gut. Therefore, it may be expected that the culture system used has a significant influence in determining the gut microbial community. In this sense, Viver et al. [48] recently documented that the gut microbiome is nonstable and highly dependent on the quality of the food. In this context, several studies showed that bioflocs are abundant and more diverse in bacterial communities compared to the gut of aquatic animals (fish and shrimp) in biofloc systems; thus, different carbon sources can influence bacterial community structure in both bioflocs and the fish gut [49,50]. As Kumar recently revised [51], biofloc particles may promote immune response by means of providing a wide range of immunostimulatory effects against microbial infections due to the presence of either lipopolysaccharides, glucans, or peptidoglycans from heterotrophic microbial cell walls. Furthermore, microbial communities growing in bioflocs have also been described to promote the host’s growth by enhancing feed digestion, as well as the function and condition of the gastrointestinal tract.

The analyses of the proximate composition of bioflocs and their lipid class composition are shown in Table 3 and Table 4, respectively. In particular, total crude protein content was always very high and increased throughout the rearing time, probably due to the presence of particles of unfed feed pellet in the water as well as changes in the composition of the microbial and invertebrate composition of biofloc particles. The same can be said regarding biofloc lipid content, keeping in mind that most of the lipids in the biofloc system were triglycerides with a very high amount free fatty acids that indicated catabolism of these nutrients [52].

The present results indicated that the biochemical composition of the biofloc particles was mostly influenced by feed residues and faecal production by the elvers, whereas the increasing content of carbohydrates in bioflocs over elvers’ farming time was mainly due to the addition of sugar to the biofloc in order to promote bacterial growth. Furthermore, Vinatea et al. [27], using a similar approach with tench (*Tinca tinca*) and flathead grey mullet (*Mugil cephalus*) as that used in the current study (i.e., similar experimental facilities, water source, and rearing conditions), found that the proximate composition of the biofloc was different in comparison to the current study. In the former trial, biofloc particles had a lower content of protein and different lipid profiles, which may be attributed to the different microbial and invertebrate composition of the biofloc, as well as differences in the proximate composition of compound feeds. In this study, the presence of digalactosyldiacylglycerols in the biofloc, especially at the start and in the middle of the trial, indicated the presence of some microalgae in the biofloc that did not appear at the end of the trial; furthermore, the high content of neutral lipids indicated the high content of feed and faeces.

Concerning elvers’ performance, results in terms of somatic growth and survival are shown in Table 5. Similar results in terms of growth performance and elvers’ survival were found in the current study when compared to elvers reared in RAS units [26,30,53,54], which indicated that this species and stage of development adapted well to biofloc systems, especially to the high levels of TSS in the water. The proper results in terms of growth performance and body condition (Fulton’s condition factor) suggested that the presence of large amounts of suspended solids did not interfere with the palatability of the feed or the olfactory sensitivity of the eels [26,54]. The present results suggested that the organic material produced within the biofloc system did manage to meet the nutritional requirements of elvers and supported their growth [26,30,53], and they confirmed the benefit of biofloc particles in terms of fish growth performance and condition [18,19,28,51].

In this sense, apparent FCR values found in the present study (1.05 ± 0.09) were similar to those reported by Hirt-Chabbert et al. [30] in RAS units (1.02–1.17) in the same experimental facilities. These results differed from other studies conducted in omnivorous species, such as tench [27], flathead grey mullet [27], tilapia (*Oreochromis niloticus*) [55], common carp (*Cyprinus carpio*) [56], and goldfish (*Carassius auratus*) [57] raised in BFT systems. For instance, apparent FCR values in *T. tinca* and *M. cephalus* were 1.75 and 2.8 times higher than in RAS, which Vinatea et al. [27] attributed to the species-specific habits of each species as well as to its adaptability to this rearing technology. Regarding tilapia (*O. niloticus*), FRC values in fish fed in BFT were 1.2 times lower than those of their congeners reared in RAS (FCR = 0.83 vs. 0.97, respectively) [55], whereas a similar trend was found in common carp (*C. carpio*) when reared in both systems (FCR in BFT ranged between 1.39 and 1.57 vs. FCR values in RAS of 1.65) [56]. Concerning goldfish (*C. auratus*), no differences in FCR were found between both rearing technologies (FCRa = 3.76–3.79) [58]. Such differences in species-specific responses of omnivorous fish in terms of feed efficiency may be related to different experimental BFT rearing conditions and management practices (i.e., TSS levels, nutritional quality of biofloc, C/N ratio, and water quality, among others) [27,53,57,59,60]. Regarding growth performance, under current experimental conditions, the coefficient of variation of the elvers’ final body weight (CV = 42.0 ± 10.6%) was higher than that previously reported in RAS units (CV = 10–26%) [30]. Size heterogeneity is common in eel farming, which has been described to affect the performance of the rearing process, since it requires a non-negligible amount of labour in size-grading activities [61,62]. The consequence of size dispersion is the so-called hierarchical size effect, which is responsible for the establishment of a group of dominant fishes that do not allow smaller (subordinate) fish to feed normally. Therefore, the largest specimens are expected to obtain the largest feed amount, grow fastest, and have the highest weight [63]. Regardless of water turbidity generated by the biofloc system that may have resulted in lesser luminosity within the BFT tanks than generally occurring in RAS systems [26,30,55], the present results indicated that tank luminosity was not low enough to reduce the hierarchical size effect, as in Rodriguez et al. [54] when rearing European eel elvers with short light photoperiods. Furthermore, the higher CV in elvers’ body weight at the end of the current trial when compared to other studies that focused on rearing this stage of eel development in RAS may be also interpreted as not all elvers having adapted similarly to BFT conditions, which suggests that further research is needed for proper optimization of elver rearing in BFT systems in order to entangle the potential effect of this rearing system on elvers’ body size dispersion.

A sudden mortality that ranged between 9.1 and 32.9%, depending on the tank replicate, occurred in the BFT tanks a few days before the trial finished. Elvers’ mortality observed was not attributed to the cultivation system itself, because all the parameters of water quality were within the recommended ranges for the species. The dead specimens presented a heavy infestation of the gills with *Gyrodactylus anguillae*, which was identified following guidelines provided by Grano-Maldonado [64]. According to Mellergaard and Dalsgaard [65], this monogenean infection can be considered the main health problem in intensive eel farming, and it is recommended to disinfect the juveniles using formalin baths prior to cultivation, as wild animals used for eel farming are usually infected by this monogenean [66]. Thus, a deficient prophylaxis of the animals before starting the experiment or the absence of a pest control system, as is the case in recirculating systems equipped with ultraviolet filters and ozone, can be considered the main causes of this mortality event. Although the application of mebendazole (1 mg/L) is efficient for eliminating this parasite [67,68], this treatment was not carried out due to the increase in mucus production that mebendazole is well-known to provoke [67] and the unknown effects that this substance could have on the microbiota of the bioflocs. The differences in parasite infestation observed among replicates may be attributed to differences in the initial parasite load among eels used; however, authors also hypothesized that these differences may be due to a stress factor that might have accentuated the chances of disease in elvers [26]. For instance, under current experimental conditions, the first replicate tank (R1) was located near the doors that give access to the room where the experiment was performed, whereas the second replicate tank (R2) was in the middle of the set-up, and the third replicate tank (R3) was placed at the far end away from the door (and the transit of people), where it was more protected from the influence of external factors. It is known that the presence of people near the culture tanks represents a stress factor for fish [69,70,71]. Considering that the position of the tanks could have caused different degrees of stress that could not be monitored, each tank received nominal values: R1 stress equal to 3 (very exposed), R2 stress equal to 2 (moderately exposed), and R3 stress equal to 1 (a little exposed). Then, by means of a linear regression, it was possible to measure the influence that the supposed degree of stress (position of the tank) had in each one of the zootechnical parameters measured in the current study (i.e., BW, TL, SGR, K, and survival) (Table 6). The assumption that stress affected the zootechnical parameters of eels in this experiment is supported by various works that deal with the subject [72,73]. In this sense, Boerrigter et al. [74] demonstrated the susceptibility of *A. anguilla* in relation to the stress caused by external factors like handling during transport and showed that eel manipulation caused an increased demand for metabolic energy during the period when animals were subjected to stress, which compromised eel’s growth. The current results were in agreement with current data that showed that the potential stress derived from the position of the replicate tank in the experimental set-up had a negative effect on elvers’ growth, condition, and survival, as shown in Table 6.

Table 7 shows a summary of the monthly cost (EUR/m^3^) of producing 1 kg of European eel elvers under BFT compared to RAS, which was estimated from Hirt-Chabbert et al. [30]. In particular, monthly running costs of rearing elvers in BFT systems compared favourably to conventional RAS units, indicating that BFT is more profitable than RAS for European eel farming at the elver stage. In this sense, different studies reported a productivity of 0.6 kg/m^2^ when eels were cultivated in earthen ponds for 18 months, whereas yield values increased up to 15 kg/m^2^ when concrete tanks with aeration were used for 12 months. However, production yields dramatically increased up to 30 kg/m^3^ in simple RAS (8 months) and up to 150 to 180 kg/m^3^ in super intensive RAS (8 months) [39,61,75]. It remains to be known if the productivity in BFT in an on-growing cycle can be equal to or exceed the productivity obtained in a simple RAS (30 kg/m^3^), to compare with what has already been achieved with tilapia *Oreochromis niloticus* [21,76].

## 4. Conclusions

The present results indicated that the biochemical composition of the biofloc particles was mostly influenced by feed residues and faecal production by the elvers, whereas the increasing content of carbohydrates in bioflocs throughout the elvers’ farming time was mainly due to the addition of sugar to the biofloc in order to promote bacterial growth. Regarding key performance indicators associated with growth and feed efficiency, the results obtained in this research allow us to conclude that European eel elvers adapted well to BFT systems regardless of the high levels of TSS in tanks as data on growth performance and FCR indicated, which compared favourably to standard RAS procedures. Furthermore, results from the first study evaluating the feasibility of using BFT with European eel at the elvers stage showed that it is possible to cultivate elvers at 2 kg/m^3^ using BFT, whereas production costs using BFT were lower than conventional RAS units. However, to make the application of this technology viable on a large scale, measures should be taken to prevent the emergence of opportunistic pathogens and, if these arise, to verify the effect that potential therapeutic substances would have on the microbiota of the biofloc.

## Figures and Tables

**Table 1:** Water quality parameters, frequency, and analytical methods used for evaluating BFT system for rearing European eel (*Anguilla anguilla*) elvers.

Parameter	Frequency	Method
Temperature (°C)	Daily	Oximeter DO 450, Eutech instruments
Dissolved oxygen (mg/L)	Daily	Oximeter DO 450, Eutech Instruments
pH	Daily	pH Multi 9310, WTW
Ammonium (mg/L) colorimetry	Daily	Nessler, MColortest^TM^, 0.05–0.8 mg/L NH₄⁺
Nitrite (mg/L)	Daily	Sulfanilamide, MColortest^TM^, 0.025–0.5 mg/L NO₂⁻
Total suspended solids (mg/L)	2 Days	Gravimetry, 100 °C (APHA 2005-2540 Y)
Ammonium (mg/L) indophenol	1 × Week	Strickland and Parsons [29]
Nitrate (mg/L)	1 × Week	JBL Test NO₃, 0.5–250 mg/L NO₃⁻
Alkalinity (mg/L)	1 × Week	Titration (APHA 2005-2320 B)

**Table 2:** Water quality parameters during the growth of European eel (*Anguilla anguilla*) elvers using biofloc technology (BFT). Data were calculated using mean daily values recorded from each replicate tank (n = 3) over a period of 60 days.

Water Parameter	BFT
Temperature (°C)	21.02 ± 1.0
Dissolved oxygen (mg/L)	7.47 ± 0.63
pH	8.06 ± 0.19
Total ammonia nitrogen (mg/L)	1.31 ± 0.98
NH_4_^+^ (mg/L)	0.039 ± 0.029
NO_2_⁻ (mg/L)	0.046 ± 0.06
NO₃⁻ (mg/L)	bdl
Total suspended solids (mg/L)	247.6 ± 81.7
Alkalinity (mg/L)	235.0 ± 18.0

Abbreviation: bdl, below detection limits.

**Table 3:** Biochemical composition (percentage in dry weight, DW) of biofloc particles from European eel (*Anguilla anguilla*) elvers culture at three different periods along the trial. Different letters within the same column indicated statistically significant differences among sampling times (ANOVA, *p* < 0.05).

	Ash (%)	Protein (% DW)	Carbohydrates (% DW)	Lipids (% DW)
Start (October)	10.72 ± 0.23 a	45.12 ± 1.21	14.99 ± 1.93 a	4.75 ± 0.08 a
Mid (November)	14.05 ± 4.74 b	46.73 ± 0.83	18.32 ± 1.83 b	4.31 ± 0.02 b
End (December)	8.36 ± 0.61 c	46.06 ± 1.70	24.05 ± 1.43 c	5.02 ± 0.03 c

**Table 4:** Lipid class composition of biofloc particles (% of total lipids) from European eel (*Anguilla anguilla*) elvers culture in a BFT system.

	Biofloc Phospholipid Content					
Sampling point	**PC**	**PS + PI**	**PG + SQDG**	**PE**	**DGDG**	**ΣPL**
Start (day 4)	8.7 ± 0.37 b	1.7 ± 0.20 b	6.1 ± 0.41 a	2.8 ± 0.30 b	1.1 ± 0.35 a	20.4 ± 0.73 a
Mid (day 32)	4.2 ± 0.20 c	7.8 ± 0.50 a	2.9 ± 0.87 b	0.8 ± 0.05 c	15.6 ± 1.24 b	12.8 ± 0.02 b
End (day 60)	10.8 ± 0.89 a	1.8 ± 0.43 b	3.1 ± 1.05 b	7.9 ± 1.08 a	nd	23.8 ± 3.04 a
	**Biofloc Neutral Lipid Content**					
Sampling point	**CHOL**	**FFA**	**TAG**	**SE + W**		**ΣNL**
Start (day 4)	13.88 ± 0.41 a	23.87 ± 0.77 b	25.93 ± 1.27 a	15.93 ± 1.06 b		79.61 ± 0.73 b
Mid (day 32)	12.79 ± 0.02 b	39.27 ± 1.22 a	15.38 ± 0.93 b	16.97 ± 0.57 b		84.42 ± 1.24 a
End (day 60)	13.51 ± 0.71 ab	15.91 ± 1.33 c	23.94 ± 2.93 a	22.88 ± 2.06 a		76.24 ± 3.04 b

Abbreviations: PC, phosphatidyl choline; PS + PI, phosphatidyl serine and inositol; PE, phosphatidyl ethanolamine; DGDG, digalactosyldiacylglycerol; PL, phospholipids; CHOL, cholesterol; FFA, free fatty acids; TAG, triacylcglycerols; SE + W, sterol esters and waxes; NL, neutral lipids; nd, not detected. Different letters within the same column denote the existence of statistically significant differences along the sampling points (ANOVA, *p* < 0.05).

**Table 5:** Final weight, final weight variation coefficient (CV), total length, specific growth rate (SGR), Fulton’s condition factor (K), survival, food conversion (FCR), and biomass gain for European eel (*Anguilla anguilla*) elvers cultivated in biofloc technology (BFT). Data are presented as the individual value for each tank replicate (R) and their average.

Parameter	R	BFT	Mean Value
Final weight (g)	1	7.25 ± 3.90	7.69 ± 0.65
	2	7.38 ± 2.45	
	3	8.44 ± 3.29	
CV final weight (%)	1	53.79	41.99 ± 10.62
	2	33.20	
	3	38.98	
Total length (cm)	1	16.50 ± 2.73	16.88 ± 0.45
	2	16.77 ± 1.77	
	3	17.38 ± 2.17	
SGR (%/day)	1	1.38 ± 0.34	1.48 ± 0.13
	2	1.43 ± 0.50	
	3	1.62 ± 0.59	
Condition factor (K)	1	0.146 ± 0.02	0.151 ± 0.004
	2	0.152 ± 0.01	
	3	0.154 ± 0.01	
Biomass gain (kg/m^3^)	1	1.59	1.85 ± 0.26
	2	1.84	
	3	2.11	
FCRa	1	1.14	1.05 ± 0.09
	2	1.05	
	3	0.96	
Survival (%)	1	67.1	77.8 ± 12.1
	2	75.4	
	3	90.9	

**Table 6:** Linear regression equations (*y* = a*x* + b) and correlation coefficient (R^2^) of body weight, total length, SGR, Fulton’s condition factor, and survival of European eel (*Anguilla anguilla*) elvers cultivated in a BFT system in relation to the degree of stress (score system = 1, 2, and 3) resulting from the location of the tanks with regard to the stress source.

Parameter	*y* = (Stress Coefficient) *x* + b	R^2^
Weight (g)	*y* = −0.595*x* + 8.88	0.831
Total length (cm)	*y* = −0.440*x* + 17.763	0.953
SGR (%/day)	*y* = −0.121*x* + 1.7167	0.898
Fulton’s condition factor (K)	*y* = −0.004*x* + 0.1587	0.923
Survival (%)	*y* = −11.9*x* + 101.6	0.970

**Table 7:** Monthly cost (EUR/m^3^) of labour, electric power, sugar, and balanced feed to produce 1 kg of juvenile European eel (*Anguilla anguilla*) elvers in the BFT and RAS systems. Data from RAS systems are estimated according to Hirt-Chabbert et al. [30].

Item	BFT	RAS	BFT/RAS
Labour	55.1	101.8	0.54
Power	3.40	105.4	0.03
Sugar	2.51	0	2.51
Feed	2.70	3.04	0.88
Total	63.7	210.2	0.31

## Data Availability

Data are available on request to the corresponding author.
